# Developing reproducible bioinformatics analysis workflows for heterogeneous computing environments to support African genomics

**DOI:** 10.1186/s12859-018-2446-1

**Published:** 2018-11-29

**Authors:** Shakuntala Baichoo, Yassine Souilmi, Sumir Panji, Gerrit Botha, Ayton Meintjes, Scott Hazelhurst, Hocine Bendou, Eugene de Beste, Phelelani T. Mpangase, Oussema Souiai, Mustafa Alghali, Long Yi, Brian D. O’Connor, Michael Crusoe, Don Armstrong, Shaun Aron, Fourie Joubert, Azza E. Ahmed, Mamana Mbiyavanga, Peter van Heusden, Lerato E. Magosi, Jennie Zermeno, Liudmila Sergeevna Mainzer, Faisal M. Fadlelmola, C. Victor Jongeneel, Nicola Mulder

**Affiliations:** 1 Department of Digital Technologies, University of Mauritius, Reduit, Mauritius; 2 Australian Centre for Ancient DNA, University of Adelaide, Adelaide, South Australia, Australia; 3 Computational Biology Division, Department of Integrative Medical Biosciences, IDM, University of Cape Town, Cape Town, South Africa; 4 School of Electrical & Information Engineering, University of the Witwatersrand, Johannesburg, South Africa; 5 Sydney Brenner Institute for Molecular Bioscience, University of the Witwatersrand, Johannesburg, South Africa; 6 South African National Bioinformatics Institute, University of the Western Cape, Bellville, Cape Town, South Africa; 7 Natural Sciences, University of the Western Cape, Bellville, Cape Town, South Africa; 8 Institut Pasteur De Tunis, University Tunis El manar, Tunis, Tunisia; 9 Institut Superieur des Technologies Medicales de Tunis, University Tunis El manar, Tunis, Tunisia; 10 Center for Bioinformatics & Systems Biology, Faculty of Science, University of Khartoum, Khartoum, Sudan; 11 Department of Electrical & Electronic Engineering, Faculty of Engineering, University of Khartoum, Khartoum, Sudan; 12 Genomics Institute, University of California, Santa Cruz, California, USA; 13 Common Workflow Language project, Software Freedom Conservancy, New York City, NY USA; 14 Institute for Genomic Biology, University of Illinois at Urbana-Champaign, Urbana, Illinois, USA; 15 National Center for Supercomputing Applications, University of Illinois at Urbana-Champaign, Urbana, Illinois, USA; 16 Centre for Bioinformatics and Computational Biology, Department of Biochemistry, Genetics and Microbiology, University of Pretoria, Pretoria, South Africa; 17 Wellcome Trust Centre for Human Genetics, University of Oxford, Oxford, UK; 18 Radcliffe Department of Medicine, University of Oxford, Oxford, UK

**Keywords:** Workflows, Pipeline, Bioinformatics, Africa, Genomics, Docker, Reproducibility

## Abstract

**Background:**

The Pan-African bioinformatics network, H3ABioNet, comprises 27 research institutions in 17 African countries. H3ABioNet is part of the Human Health and Heredity in Africa program (H3Africa), an African-led research consortium funded by the US National Institutes of Health and the UK Wellcome Trust, aimed at using genomics to study and improve the health of Africans. A key role of H3ABioNet is to support H3Africa projects by building bioinformatics infrastructure such as portable and reproducible bioinformatics workflows for use on heterogeneous African computing environments. Processing and analysis of genomic data is an example of a big data application requiring complex interdependent data analysis workflows. Such bioinformatics workflows take the primary and secondary input data through several computationally-intensive processing steps using different software packages, where some of the outputs form inputs for other steps. Implementing scalable, reproducible, portable and easy-to-use workflows is particularly challenging.

**Results:**

H3ABioNet has built four workflows to support (1) the calling of variants from high-throughput sequencing data; (2) the analysis of microbial populations from 16S rDNA sequence data; (3) genotyping and genome-wide association studies; and (4) single nucleotide polymorphism imputation. A week-long hackathon was organized in August 2016 with participants from six African bioinformatics groups, and US and European collaborators. Two of the workflows are built using the Common Workflow Language framework (CWL) and two using Nextflow. All the workflows are containerized for improved portability and reproducibility using Docker, and are publicly available for use by members of the H3Africa consortium and the international research community.

**Conclusion:**

The H3ABioNet workflows have been implemented in view of offering ease of use for the end user and high levels of reproducibility and portability, all while following modern state of the art bioinformatics data processing protocols. The H3ABioNet workflows will service the H3Africa consortium projects and are currently in use. All four workflows are also publicly available for research scientists worldwide to use and adapt for their respective needs. The H3ABioNet workflows will help develop bioinformatics capacity and assist genomics research within Africa and serve to increase the scientific output of H3Africa and its Pan-African Bioinformatics Network.

## Background

Computational biology has shifted significantly since the introduction of high-throughput sequencing and genotyping platforms [1]. Processes that were previously slow and research exclusive tasks, have become routine applications in day-to-day operations in bioinformatics and medical genomics. These advances have resulted in a biomedical data deluge with sequencing centres routinely generating data in the petabyte scale, leaving researchers and clinicians with a data processing and analysis bottleneck. Moreover, the need to reproduce results both internally and by others, and sharing of complex computational analysis workflows, is a requirement for good scientific practice [2, 3]. Thus, automated reproducible workflow-based data processing has become a necessity for the advancement of genomic research and its applications.

Using powerful workflow management systems not only facilitates high throughput analysis, but also allows the scientists to automate tedious repetitive tasks of managing the data, running the different tools, managing the intermediate files, and dealing with job schedulers [4, 5]. Modern workflow management systems offer a high level of reproducibility, portability and computing platform independence enabling researchers to focus more on developing new methods and the interpretation of the results.

### Motivation

The Human Heredity and Health Consortium (H3Africa) was launched in 2011 by the African Society for Human Genetics, the US National Institute of Health and the Wellcome Trust to promote and develop the capacity for genomics research in Africa [6]. The H3Africa consortium comprises over 20 research projects, the Pan-African Bioinformatics Network for H3Africa (H3ABioNet) and three bio-repositories. H3ABioNet [7] is building capacity for bioinformatics within Africa while supporting the H3Africa projects with their data management, analysis and bioinformatics requirements. The need for automating the key workflows required for H3Africa is particularly acute due to the volume of genomics data generated. Besides the general importance of reproducible and reliable workflows, H3Africa funding requires that after a short embargo period all data generated by H3Africa projects must be placed in the public domain. It is important that African scientists operating in resource-scarce environments are provided with the tools, ability and capacity to analyse African genomics data as equal partners rather than data generators, and be able to compete effectively with larger and better resourced groups.

For these reasons, H3ABioNet took the strategic decision to organize a hackathon for developing four workflows to support the major types of analyses that H3Africa groups will require, before the bulk of the H3Africa data is generated. We identified the most important analyses as (1) variant calling on next generation sequence (NGS) data; (2) 16S rDNA sequence analysis for metagenomics; (3) genome-wide association studies; and (4) imputation. In the future these workflows will need to be customized to the type of analysis, adapted to use new software packages, and also other workflows will need to be created. Therefore, an important subsidiary goal was to develop capacity for portable, reproducible workflow building in Africa. An important constraint on this development was that the workflows should be as portable as possible, given the heterogeneous computing environments of the different groups e.g High Performance Computing (HPC) centers, University and lab clusters, and cloud environments, where accessible. The use of container technology (Docker, in our case) was considered crucial, though the workflows do not rely solely on Docker, as many high-performance computing clusters offer alternative implementations such as Shifter (https://github.com/NERSC/shifter) or Singularity (https://www.sylabs.io/docs/). This paper reflects on the technical aspects and details of implementing these workflows, and the lessons learned. The produced workflows are flexible, robust, portable for heterogeneous computing environments and use current best practices in bioinformatics.

## Implementation

### Workflow management

There are various workflow systems and languages such as Nextflow, and open community standards for expressing workflows such as Common Workflow Language (CWL) [4]. For the purpose of developing the H3ABioNet workflows, we chose existing community workflow standards or workflow systems with languages commonly in use by the bioinformatics community, and in which there were existing skills within H3ABioNet and its collaborators.

#### Common workflow language (CWL)

The Common Workflow Language (CWL) is a workflow description standard designed with a focus on portability, easy tool and workflow definitions, and reproducibility of data-intensive analysis workflows [8]. CWL aims to be an industry standard in the field of bioinformatics and other data intensive sciences. CWL consists of specifications that can be used by data scientists to implement powerful workflows [8]. CWL relies on technologies including JSON-LD, Avro for data modeling, and Docker-compatible software container runtimes (e.g. Singularity and uDocker) for portability.

CWL has been developed by a multi-vendor working group consisting of various organizations and individuals with an interest in portability of data analysis workflows. The CWL standards are actively used by leading institutions, such as the Wellcome Trust Sanger Institute (https://www.sanger.ac.uk), Institut Pasteur (http://www.pasteur.fr), and UCSC (http://ucsc.edu). As of submission of this paper there are several execution engines that support workflows written in CWL: Arvados, Toil, Rabix, and CWL-Airflow. Additionally, CWL is in the process of being adopted by popular bioinformatics workflow management systems, such the Galaxy Project [9], Rabix [10], AWE [11], and others. Workflows A and B below were implemented using CWL.

#### Nextflow

Nextflow [12] is a workflow language and system developed at the Centre for Genome Regulation in Barcelona. Although it has a few specific built-in features to support bioinformatics, it is a general-purpose workflow system that runs on Unix-like systems including Linux and MacOS. The language is a domain-specific language built on Groovy. Nextflow supports: execution of workflows, with partial resumption; containerisation with Docker and Singularity; and multiple execution modes including local execution, execution on clusters, Amazon EC2 (cloud-init and AWS Batch), Kubernetes, and OpenStack. The Nextflow workflows are highly portable, e.g. a scientist can run the same workflow:

- on a dedicated computer with all the underlying application software installed;
- distributed across a compute cluster (again assuming all underlying application software has been installed): the Nextflow program submits the necessary jobs to the cluster job manager on the scientist’s behalf, considering dependencies between tasks; the Nextflow monitoring process itself will run on the head node;
- without any of the bioinformatics software installed on the system, and they are set up using Nextflow’s Docker support (locally, on a Docker Swarm or on Amazon EC2).

In all cases Nextflow adapts its execution strategy to the environment. Workflows C and D below were implemented using Nextflow. We provide documentation on what bioinformatics software needs to be installed, as well as a set of Docker images so that the workflows can be run without installing any software other than Nextflow and Java.

### **Workflow A: whole Genome/Exome NGS data analysis**

Whole genome and whole exome shotgun sequencing has become an essential tool for research and medical applications [13–15]. The H3Africa projects will be generating exome and whole genome sequence data, hence implementing an automated variant calling data analysis workflow for analysis of African genomic data is essential. The H3ABioNet hackathon participants opted to implement an extended version of the Broad Institute’s Genome Analysis ToolKit (GATK) [16] Best Practices version 3.5 [17, 18]. This GATK best practices for variant calling has been extensively validated and accepted as an industry standard for human NGS data analysis [18]. The workflow (Fig. 1) was extended during the H3ABioNet hackathon to include:

1. Sequencing adaptor trimming with Trimmomatic [19], which provides extensive short read trimming, including sequencing adaptors and barcode trimming, and base quality based trimming.

**Fig. 1:**
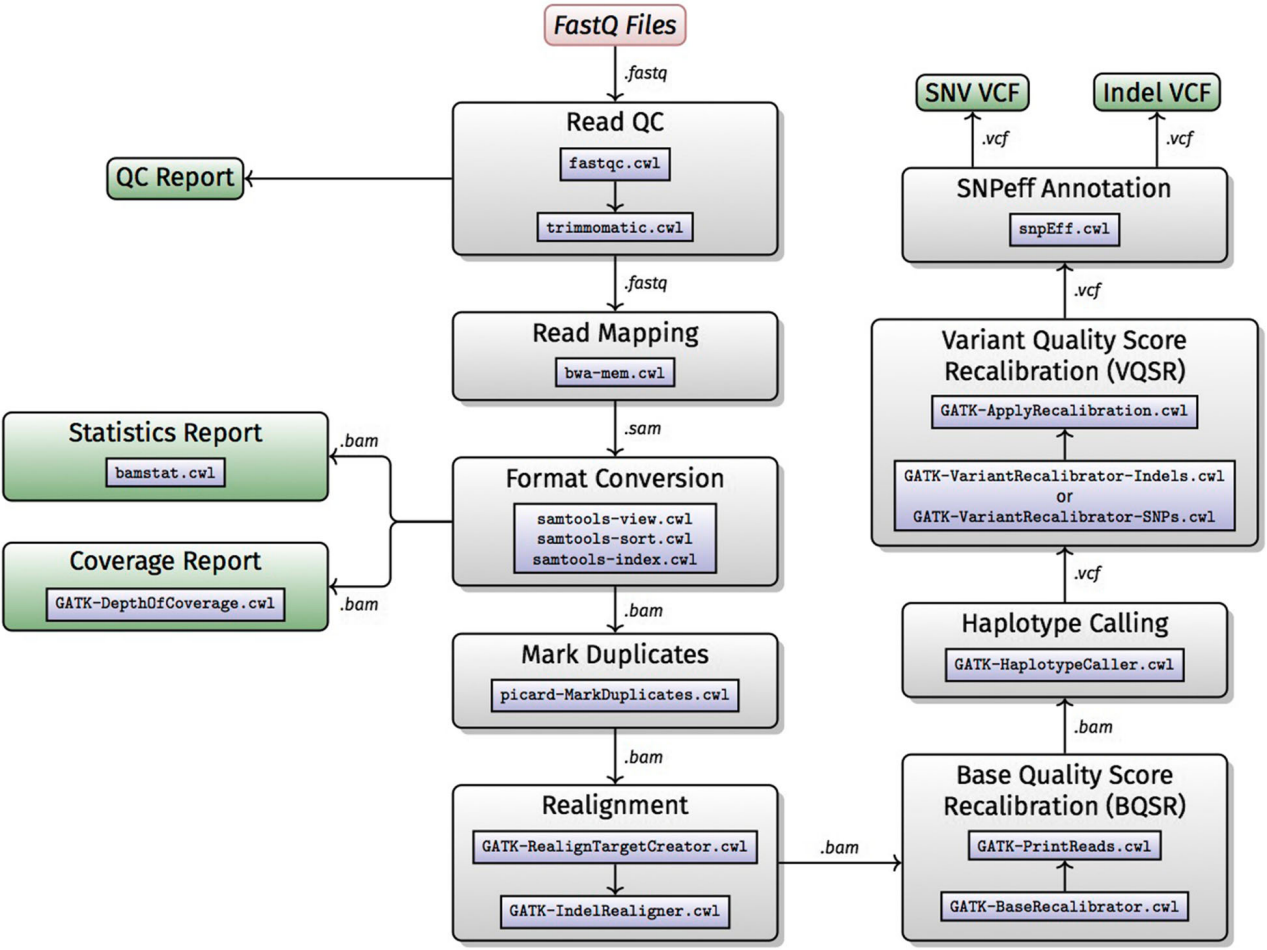
Workflow A: whole genome/Exome NGS data analysis
2. Quality control (QC) of the input fastq files with FastQC (https://www.bioinformatics.babraham.ac.uk/projects/fastqc/), which provides an exhaustive range of QC, allowing the user to diagnose frequent caveats and issues occurring before (e.g. library preparation), after the sequencing (e.g. tile issues), as well as post-sequencing (e.g. demultiplexing and adaptor trimming).
3. Short reads mapping: BWA-MEM is used to perform paired-end mapping of Illumina reads [20]. BWA-MEM is the industry standard.
4. QC of the aligned reads: BAMStats (http://bamstats.sourceforge.net) provides a comprehensive overview on mapping quality and presents the results in a detailed report. Such a report provides extremely useful screening for mapping issues.
5. QC of the aligned reads using GATK’s DepthOfCoverage tool [16] to ensure the observed depth of coverage meets expected yield values.
6. Indels and single nucleotide variant (SNV) annotation: SnpEff [21] extends the VCF file containing the variants with information relevant for downstream analysis. The information included ranges from the SNP rsID, to clinically relevant variants from ClinVar [22].

The workflow takes advantage of Docker integration with CWL to handle the tools’ dependencies, with the exception of GATK, where the user has to provide their own GenomeAnalysisTK.jar file as one of the workflow inputs for licensing reasons. The workflow is available on the CWL workflows library on GitHub: https://github.com/h3abionet/h3agatkand the container at Dockstore: https://dockstore.org/workflows/github.com/h3abionet/h3agatk.

### **Workflow B: 16S rDNA diversity analysis**

**Fig. 2:**
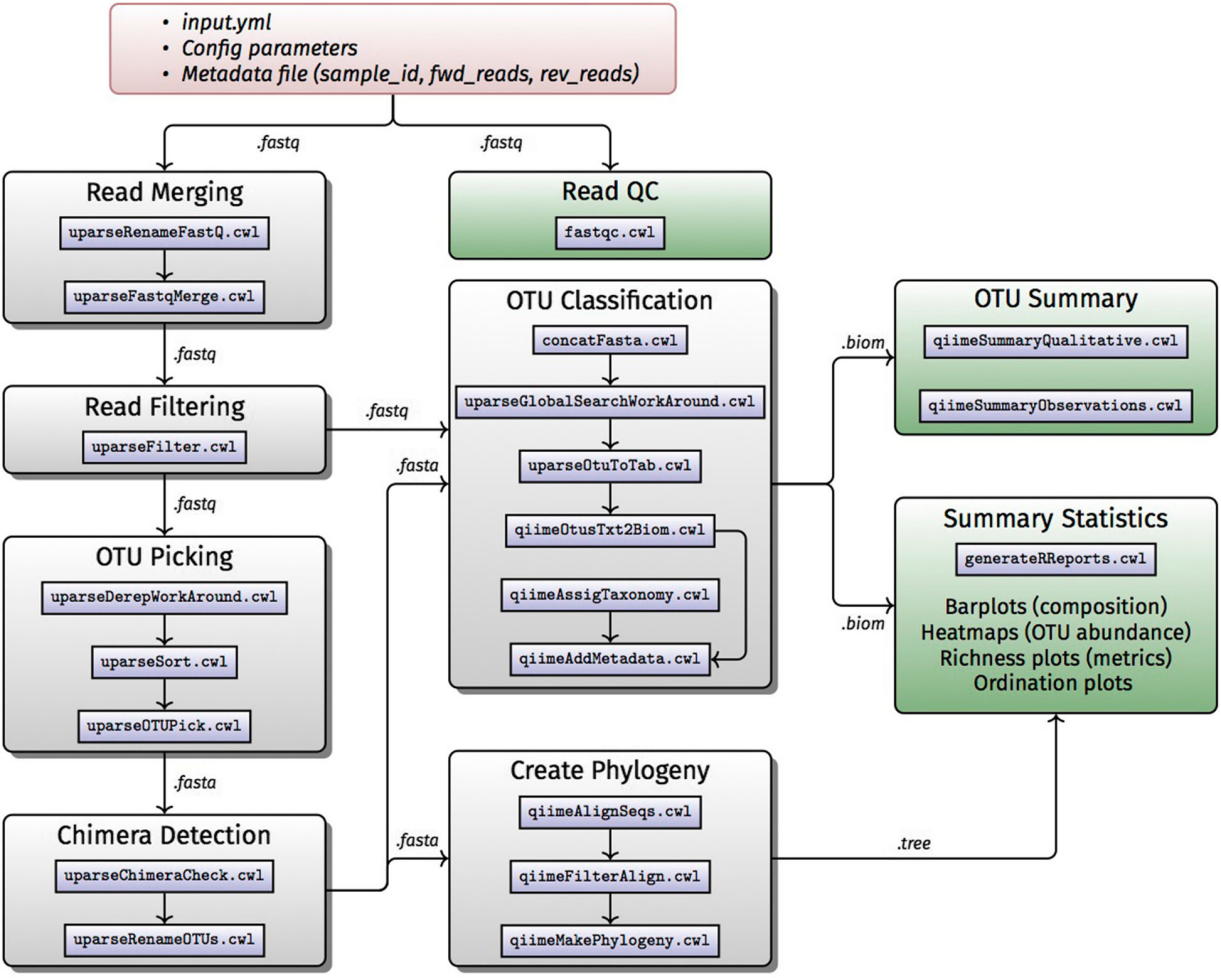
Workflow B: 16S rDNA diversity analysis
  (https://www.bioinformatics.babraham.ac.uk/projects/fastqc/). The reports can give guidance on which QC setting needs to be modified for downstream processing.
- Reads are prepared to have naming that is compatible with USEARCH (http://www.drive5.com/usearch). We use fastq_renamer to accomplish this.
- Paired end reads are merged together using USEARCH.
- Low quality merged reads are removed using USEARCH.
- Merged reads are clustered together into OTUs based on similarity derep_workaround, fasta_splitter and using USEARCH.
- Chimeras are also removed in order to avoid the emergence of spurious OTUs using USEARCH.
- Taxonomic assignment of OTUs are performed using QIIME (http://qiime.org).
- A phylogenetic tree is created from the OTU sequence alignments using QIIME.
- Some descriptive statistics and plots are generated from the resulting BIOM and tree file using Phyloseq [24].

The 16S rDNA workflow (Fig. 2) was developed using CWL, for performing 16S rDNA diversity analysis of microbial species in metagenomic samples using raw sequence data generated by high-throughput sequencing of 16S rDNA gene amplicons [23]. In order to perform the complete analysis, the quality of the data has to be checked and the data must go through a number of computational processing steps. All the tools used in the complete analysis have been described using CWL to create a complete workflow which can be easily used by researchers. More specifically, the tools used can be summarized as:

- QC reports are generated using FastQC

The 16S rDNA workflow consists of several steps, each represented by one CWL workflow description, as shown in Fig. 2.

Since the workflow has been developed using CWL which already provides support for the use of Docker, all the tools have been included in the form of a Docker container. It should be noted that since USEARCH requires a license agreement, users should first go through the license application process prior to using this component in the workflow. The resulting workflow for the 16S rDNA Diversity Analysis is available on GitHub at https://github.com/h3abionet/h3abionet16S and the container at Quay.io: https://quay.io/repository/h3abionet_org/h3a16s-fastqc, https://quay.io/repository/h3abionet_org/h3a16s-in-house, https://quay.io/repository/h3abionet_ org/h3a16s-qiime and https://quay.io/repository/h3abionet_ org/h3a16s-r.

### **Workflow C: genome wide-association studies**

The H3Africa Consortium will genotype over 30k individuals using a custom designed African genotyping array. This effort will create the first large influx of data for a range of genome-wide association studies (GWAS), facilitating the need for the development of a robust and efficient workflow. GWAS data analysis is a multi-step approach beginning with several QC steps prior to the downstream association tests. A GWAS data analysis workflow (Workflow C) was therefore implemented using Nextflow. The workflow (Fig. 3) consists of 3 modules, which can be swapped in and out depending on the analysis needs:

1. Conversion from Illumina TOP/BOTTOM call format to PLINK format.

**Fig. 3:**
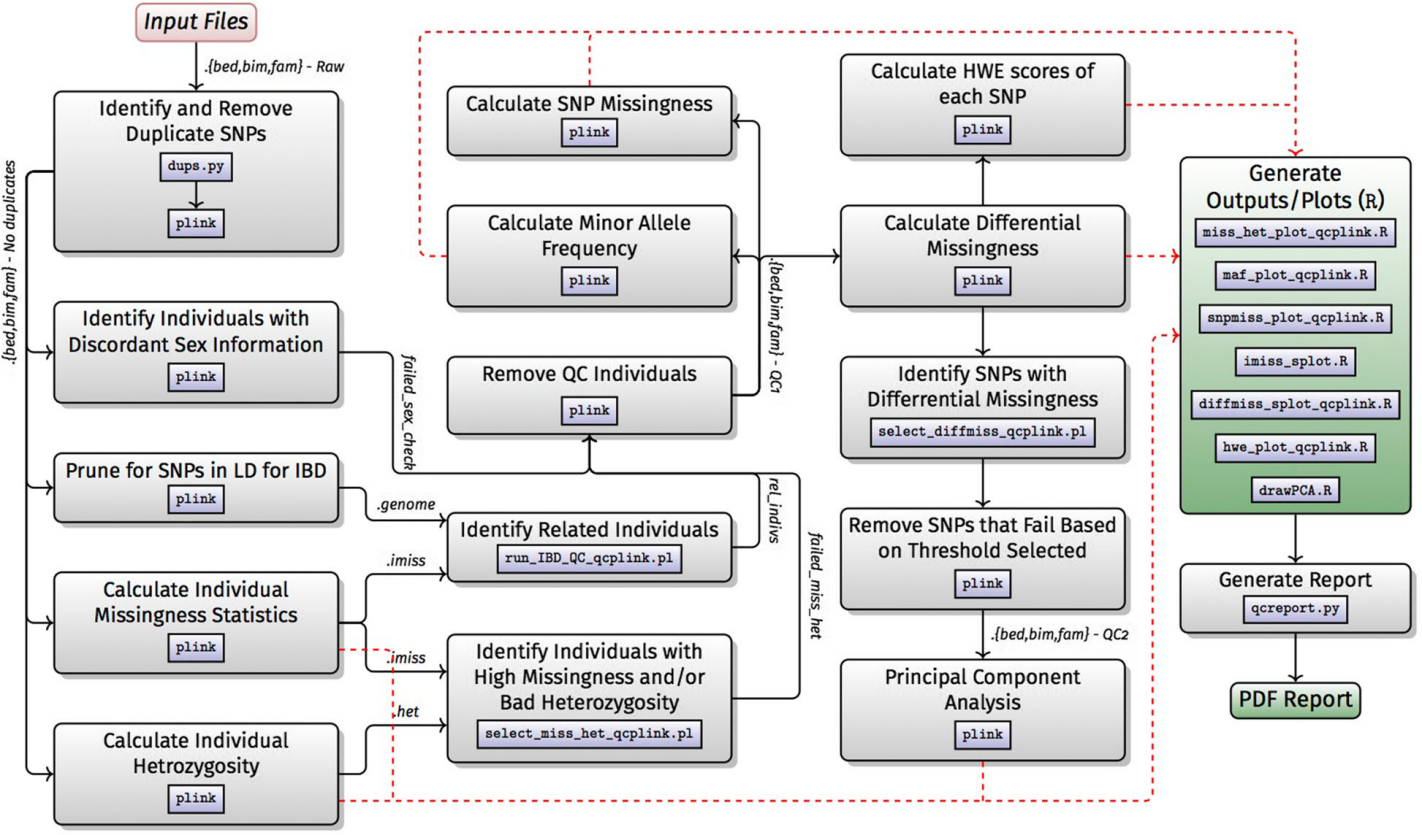
Workflow C: genome wide-association studies
2. The core workflow carries out a set of QC steps, starting with standard PLINK files and resulting in quality controlled PLINK files.
3. Basic association testing and structure analysis.

In addition, we expect many researchers will use the imputation workflow after QC and before association testing. An overview of these modules is given below.

#### Conversion from illumina TOP/BOTTOM format

We expect that most H3Africa groups will receive their raw genotype calls in Illumina TOP/BOTTOM format. These need to be converted into PLINK format whilst trying to align the calls to the correct strand. Though the actual conversion into PLINK format is trivial, the data set sizes are very large and conversion can take hundreds of CPU hours and is ideally parallelised. This step has been included in the workflow in case it is necessary.

#### Quality control

The QC workflow filters the input data using standard protocols in PLINK (e.g. [25]). It checks for discrepancies in sex information; differential missingness between cases and controls; deviation from Hardy-Weinberg equilibrium for SNPs in cases; related individuals; individuals with unusually high or low levels of heterozygosity; individuals and SNPs with high missingness; and minor allele frequency. Sensible default values are given for cut-off values, but the user can easily provide their own. A detailed PDF report is produced explaining how the QC was conducted: workflow version and parameters are recorded, as well as the MD5 sums of the input and output files. The QC report may be reviewed by the user after the initial analysis to determine if the default QC parameters are appropriate for the data being analysed. If required, these parameters may be modified in the Nextflow.config file.

#### Association testing

This workflow performs association testing on PLINK formatted files, including adjustment for multiple testing in PLINK. In addition to the basic association tests, the workflow currently supports Cochran-Mantel-Haenszel (CMH), linear and logistic regression, permutation and mixed-model association testing. The PLINK input files are also used to perform a principal component analysis (PCA) and a PCA plot is generated to identify any possible population structure in the data set. This is the workflow least amenable to general automation because analysis approach will be very dependent on phenotype, structure, GWAS test etc. For instance, it is particularly complicated to automate the management of the different population structure cases. While, for example, some users of our workflow will have a homogeneous group to study, others may have samples with significant admixture. The goal of the association testing workflow is to perform an initial analysis and review the results, allowing scientists to gain further insights into the complexity of their data before performing their own bespoke analysis. This workflow is under active development as H3A groups analyse data from round 1 of H3A. Sample runs and extensive documentation can be found at: http://github.com/h3abionet/h3agwas.

### **Workflow D: SNP imputation**

Imputation is likely to be run in the context of a GWAS, studying population structure, and admixture studies. It is computationally expensive in comparison to other GWAS steps. For this reason, we decided to develop the imputation workflow separately. This allows the two workflows to be run as an integrated whole, or running the imputation separately on a larger compute platform.

**Fig. 4:**
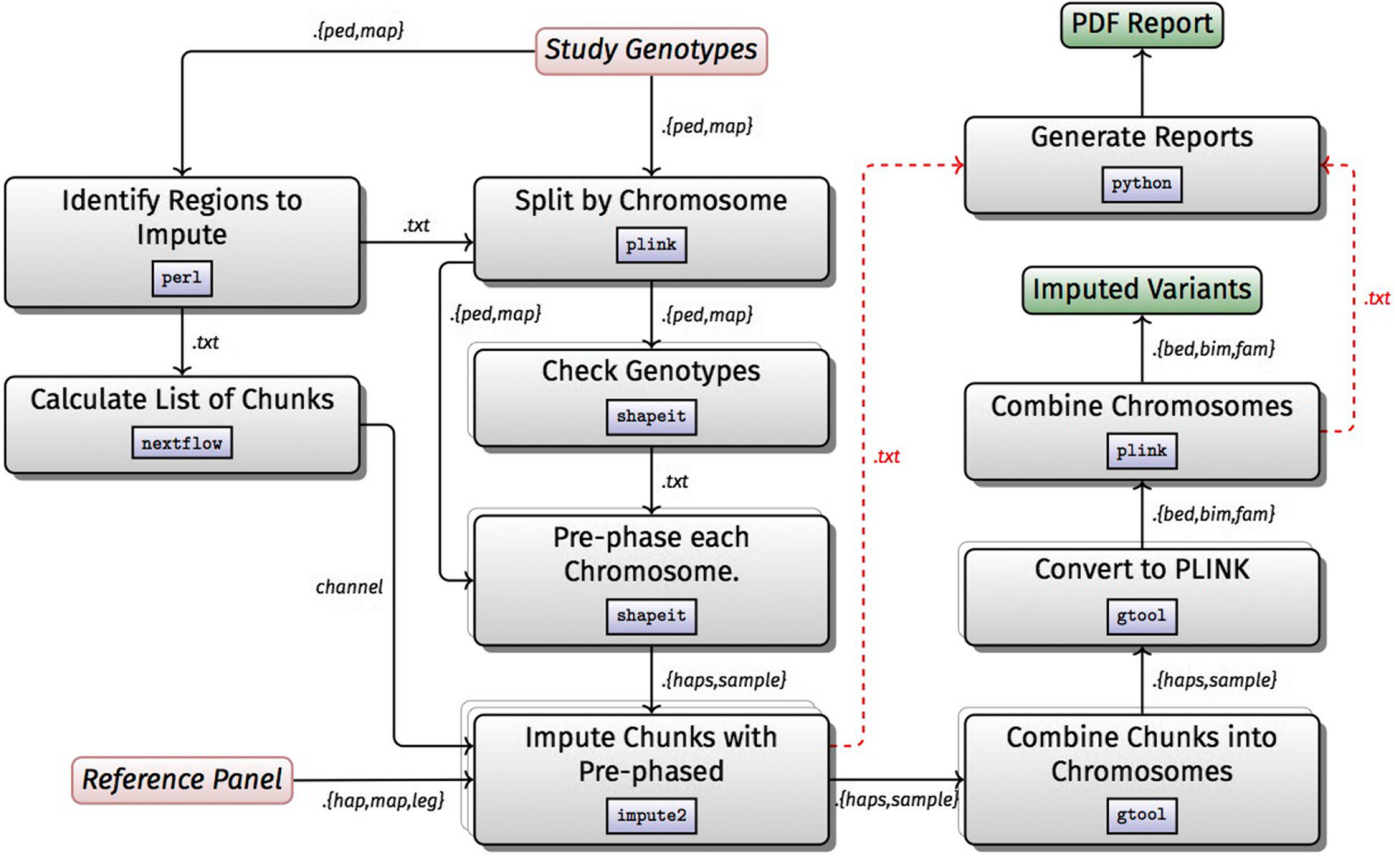
Workflow D: SNPs imputation

The workflow was developed using Nextflow, and identifies regions to be imputed on the basis of an input file in PLINK format and produces output in IMPUTE haplotype format (Fig. 4). The ped and map input files are split by chromosome using PLINK and chromosome extents are identified using a combination of awk [26] and grep [17]. Genotyped positions on individual chromosomes are checked for strand flipping errors, and improperly stranded positions are excluded using SHAPEIT [27]. Genotyped positions are then prephased using SHAPEIT in parallel on each chromosome. After prephasing, IMPUTE2 [28] is run in parallel across all 500 kB blocks in the entire genome. Imputed blocks are then combined into a single compressed haplotype file using a perl script which is provided with the workflow. Finally, we convert this file back to a PLINK dataset for integration back into the GWAS workflow.

The workflow as implemented is capable of using multiple reference panels, and has been tested in whole-genome imputation using the Haplotype Reference Consortium panel as well as the 1000 genomes panel phase 3 (which is utilized by default). Because assuring genotype and reference strand alignment is critical for proper imputation, we have also included a perl script that is capable of strand flipping user-supplied ped and map files given an Illumina TOP/BOT probe strand identification file, a subset dbSNP VCF file, and a reference genome to identify strands. After running this correction step on a typical Illumina GWAS chip, less than 0.05% of probes had strand mapping issues that had not been resolved. The workflow code and documentation can be found at: https://github.com/h3abionet/chipimputation.

## Results

Both CWL and Nextflow based workflows offer high levels of reproducibility and portability. The workflows described below are available on GitHub. All come with extensive documentation.

### Workflow A: whole Genome/Exome NGS data analysis

The workflow (https://github.com/h3abionet/h3agatk) has been tested on local machines, EGI FedCloud resources (Fernández-del-Castillo et al. 2015), AWS EC2 as well as on a Microsoft Azure VM both with and without Docker. The workflow requires Docker to be installed, and the GATK jar file and sequence reads in fastq format as input files. We tested the pipeline using several whole-exome and whole genome datasets such as the Genome in a Bottle (GIAB) NA12878 Garvin exome. For instance, the user can use the testprepare_test.sh script to set up a test environment and download the necessary data files to run GAIB NA12878 exome. Running this test example on a 16 core, 128 GB+ Ubuntu VM running on Azure machine takes less than 8 h and requires about 500 Gb of storage space available during the analysis

### Workflow B: 16S rDNA diversity analysis

This workflow (https://github.com/h3abionet/h3abionet16S) has been tested on local computers (Linux and MacOS) with and without Docker, AWS EC2 and Azure VMs with and without Docker, an SGE cluster with Docker support and a PBS cluster without Docker support. Documentation to setup the package and run the workflow has been created and can be accessed here: https://github.com/h3abionet/h3abionet16S/tree/master/workflows-cwl.

For testing we used a 16S rRNA dataset that comprised of three dogs which were treated with increased percentage of a compound in their diet on 5 levels. The total dataset had 15 samples with an average of 25,000 reads per sample and an overall size of 2.4 GB. We used the the greengenes database (3.7 GB) for classification and phylogenetic tree generation and the gold.fa “Gold” database (16 MB) for chimera checking. We ran the CWL pipeline using the cwltool and it took 36 min to complete on a single core with 8 GB of RAM. An output of 4 GB was created (excluding the CWL cache size of 4.8 GB). The final OTU file consisted of 187 OTUs. Visually inspecting the per sample diversity plot, heatmap and abundance plot pointed to difference between the 3 dog groups. Pulling the BIOM file into R and analysing the results with the MetegenomeSeq showed that there is a significant differential abundance between the dog groups. Inspecting ordination plots with Phyloseq also showed a clear difference between the groups.

### Workflow C: genome wide-association studies

This workflow (http://github.com/h3abionet/h3agwas/) has been tested on local computers (Linux and MacOS) with and without Docker, clusters running Torque (with and without Docker) and the Bright Cluster Manager (without Docker), Docker Swarm and cloud-init (http://cloudinit.readthedocs.io). Nextflow has direct support for Amazon EC2 which allows the dynamic creation of EC2 clusters using Nextflow commands. We have packaged an Amazon AMI for users to support our workflow (and would work for the other workflows). The same workflow runs across all environments, using different parameters for start-up. The workflow setup requires installation of Nextflow (a one line command) and Java 8, and either a Docker engine or the installation of the underlying application software. For those unable to use Docker, e.g., on HPC systems which do not support it, the underlying application software such as PLINK and Python will need to be installed and added to the default PATH at the moment of the execution. The workflow also supports Singularity.

The pipelines have been successfully used by three H3A groups to analyse their H3A project data. The largest analysis we know was that of the AWI-Gen group where approximately 11.5 k samples were genotyped using the H3A Genotyping array (approximately 2.2 m SNPs). The AWI-Gen project [29] is exploring genetic and environmental factors in cardio-metabolic disorders in African populations. Almost 12,000 participants have been recruited at six sites in four African countries, have been finely phenotyped and all genotyped on the H3A Genoyping arrays. All three sub-workflows were used.

Example computational costs: Reporting computational costs on real data sets in a production environment is difficult so these figures are given as indication and time will also depend on the format of the raw data provided by the genotyping centre. Running the *topbottom.nf* workflow on 106 k samples for a 2.2 m array (input data size is 390 GB of compressed call data) takes roughly 120 CPU hours, completing in 1.4 h on a well-provisioned cluster. The workflow results in 228 processes being executed The maximum peak resident set size of any single process was 9.5GB, but most of the tasks required less than 2 GB RSS. The resulting PLINK data set was roughly 5.8 GB in size. Running the *plink-qc.nf* workflow on an 11.3k sample set on the same genotyping array (7GB PLINK input data) took 1.25 h using roughly 9 CPU hours. The workflow comprised 31 processes. The largest resident set size of any process was 10 GB. The *plink-assoc* workflow cost is very data and parameter dependent – using the same data set as *plink-assoc.nf* can take anything from an hour to several days (depending on what co-variates are used, whether permutation testing is done, whether linear mixed-model are used). The workflow tries to parallelise as much as possible (e.g., using multi-core where possible, running separate analyses in parallel).

### Workflow D: SNP imputation

This workflow has been tested on local computers (Linux) with and without docker, an SGE cluster without Docker support, and an OpenStack cloud with Docker support. Documentation to set up and run the workflow is present at https://github.com/h3abionet/chipimputation.

We ran the workflow on a modest, publicly available dataset of 471 samples genotyped on the Affymetrix SNP6 array against the standard IMPUTE2 reference panel of 2504 samples (85M sites). On a 192-core cluster the workflow completed in 58 h, utilising 17 k CPU hours. 17 k processes were executed, with maximum and minimum runtimes of 1 and 250 min respectively. The final output dataset was roughly 45 GB. Maximum memory usage was 8GB across all processes. It is worth noting that earlier runs reported processes consuming 256 G RAM, a result of PLINK reserving half of a machine’s RAM by default. This was adjusted in the pipeline, but illustrates the need to refine configurations for individual environments. It also demonstrates the advantage of running in containers.

### Docker images

We have registered the Docker images for the pipelines at: https://quay.io/organization/h3abionet_org and https://dockstore.org/workflows/github.com/h3abionet/h3agatk.

## Discussion

While several workflow management systems are available, they are designed to offer a specific set of functionalities. For example, Galaxy [30] and Taverna [31] focus on providing a user friendly “point and click” graphical interface. While other platforms such as COSMOS and Pegasus, among many others, are focused on offering frameworks for a high-level of parallelisation. However, these platforms each use unique workflow definition languages, and operate at different levels of complexity. Earlier efforts to enable interoperability between different workflow definition languages was Tavaxy [32] enabling the running of workflows defined in Galaxy and Taverna. Community driven projects such as CWL offer a more robust approach towards standardisation of workflow definitions, whereas Nextflow addresses more specialised needs such as the partial resumption of a workflow without the need to restart the whole analysis. This makes it suitable for long and computational heavy, and error-prone workflows.

### Related work

Building upon the experience of Workflow A, “H3ABioNet GATK Germline Workflow”, the Human Genetics Informatics team at the Wellcome Trust Sanger Institute built “gatk-cwl-generator” which generates CWL descriptions from the GATK documentation https://github.com/wtsi-hgi/gatk-cwl-generator/. M. Crusoe used his experience helping the Workflow B team (“H3ABioNet 16S rDNA diverstity analysis”) when he assisted EMBL-EBI in converting their core Metagenomics service pipelines from in-house scripts to CWL: https://github.com/EBI-Metagenomics/ebi-metagenomics-cwl though the initial version of those CWL descriptions did not have accompanying Docker software containers, unlike the work of the Workflow B team.

There are no directly comparable workflow systems for Workflow C (GWAS). The closest for QC is the work of Ellingson and Fardo [33], which is a collection of Python and R scripts for performing QC. GenCloud [34] was a workflow for GWAS analysis using grid-based technology for authentication and running in an OpenStack environment; however, no further work has been done since 2014 and it is not available for general installation. GWASpi [35] is a Java program that provides end-to-end analysis of data. However, it has not been updated for several years, and only provides limited functionality. Several groups have developed in-house systems of great power and flexibility (e.g. the Edinburgh GWAS pipeline [Wilson and Joshi, personal communication], https://www.ikmb.uni-kiel.de/research/genetics-bioinformatics/bioinformatics/gwas-pipleline). However, these are not designed for easy porting to other environments and may not even be available to other researchers.. Our pipeline is distinguished by more general functionalities and being easier to be customised by users. The use of containers makes installation of the software *much* easier across a range of systems, and the use of Nextflow as a language also aids portability in allowing execution in a range of execution environments, and most importantly the scalability of execution as Nextflow can transparently map the workflow to the available computational resources. We have also put considerable effort to provide documentation, training material and make installation and use as easy as possible.

### Reflections on workflow building and use

The primary goal of the project was to produce out of the box operational workflows, and workflow languages used for implementation were chosen for pragmatic reasons. Discussions of the possibility of standardising on one workflow language were had, but there was a desire by different groups to use either CWL and Nextflow. The use of the two workflow languages would allow expertise and capacity in both languages to be developed within H3ABioNet and allow the exploration of different technologies.

Both infrastructures offer robust workflow development environments, and have a range of useful features and very responsive support communities. Beyond learning how to use and implement them, the H3ABioNet hackathon was not designed to evaluate them individually or compare and contrast the workflow languages. CWL and Nextflow are different in their philosophy and syntax which partly reflects their very different origins. Nextflow comes from one research group which drives the Nextflow language and system development using its community as a sounding board. CWL is a language rather than a system, and is developed by a multi-vendor community. The standards are agreed upon by the community, and tools from different vendors support it. Our tentative experience was that Nextflow is easier to learn and may fit the needs of smaller and mid-size groups better; however, these findings are based upon subjective experiences and may simply reflect that different programmers have personal preferences beyond objective justification. At the time of this hackathon none of the CWL graphical interfaces which have since been written, such as the Rabix Composer, were available.

Four members of the Hackathon group attended a Nextflow Hackathon organised by CRG in Barcelona. As part of their task for the hackathon it was decided to convert the h3abionet16S CWL workflow running on Docker containers to Nextflow using Singularity containers. Since two of the CRG hackathon members were originally part of Stream B that had developed the h3abionet16S CWL workflow, they were able to compare CWL and Nextflow based on their own experience. They felt that it was quicker to develop the h3abionet16S workflow in Nextflow, possibly because they already had a CWL template to work from. The members also consider Nextflow to be the best choice for getting a bioinformatics pipeline up and running quickly, but will consider setting up CWL workflows if the workflows need to be integrated into frameworks such as Galaxy. Nextflow, similar to major workflow systems, integrates seamlessly into common resource managers and provides an advantage of having clearly standardized code blocks that are implicitly intended for parallelisation, and code blocks that are executed sequentially which is common in designing bioinformatics workflows where inputs are dependent on outputs of prior processes. Other nice user features of Nextflow include embedded support for user notification on the progress saving the user from explicitly coding mailx commands.

Errors must be handled carefully in workflows. For CWL, configuration errors are handled by each CWL engine on their own and not typically at the workflow level. Basic type checking of inputs (File, string, number) is built in, and the CWL standard supports declaration of data stream formats, but verifying those formats beyond the users’ declaration is an optional responsibility of the engine. Likewise, one can specify what return codes mean that a temporary or a permanent error has occurred, but advanced error handling logic beyond that is specific to each CWL implementation. For example, IBM’s “CWLEXEC” for LSF has its own error handling directives. CWL users can also insert conceptual checkpoint steps that verify that the data at a certain point in the workflow meets desired parameters, forcing an early termination if it does not.

There are CWL engines that are able to submit to cluster environments, however they were not tested on the h3abionet16S CWL workflow; the CWL reference runner (cwltool) that was used in testing purposely does not provide that functionality. The Nextflow version of the h3abionet16S package is now also available in the GitHub repository (https://github.com/h3abionet/h3abionet16S/tree/master/workflows-nxf).

One complication of workflows is that exceptional events or errors may be obscure for the user. The GWAS workflow does some pre-checks of the user data; however, many errors can only be detected when the actual computation is done. Nextflow’s error messages are intended for people comfortable with Nextflow. Within these constraints we try to print meaningful error messages, and have requested modification to Nextflow to allow greater control of what errors appear.

H3ABioNet plans to make the imputation workflow available as a service to African researchers, together with an African-specific reference (software and computational resources). There are informal arrangements within the network where the better-resourced nodes make their resources available to other nodes or African researchers.

## Conclusion

In this paper we present the effort of H3ABioNet to create four workflows able to run on heterogeneous computing environments such as HPC, University clusters and cloud computing environments, addressing most of the computation needs of African researchers, and enabling automated, fast and accessible data processing. Development of such workflows will assist in providing a boost to the research output in Africa while developing capacity in bioinformatics, and also allow users from other parts of the world to use and modify the workflows for their needs. These four H3ABioNet workflows were developed using well supported, flexible and well documented workflow definition languages and are easy to upgrade and modify to fit the specific requirements of their users.

## Availability and requirements

Project name: H3ABioNetProject home page: https://github.com/h3abionetOperating system(s): Linux/Unix/Mac Programming language: Nextflow, CWLOther requirements: DockerLicense: All code for Workflows A, B, C and D are available under MIT license. Any restrictions to use by non-academics: NoneLimitations imposed by the software and packages used: None

## Abbreviations

16S: 16S rRNA
BWA: Burrows-wheeler aligner
CWL: Common workflow language
CMH: Cochran-mantel-haenszel
GATK: Genome analysis toolKit
GWAS: Genome-wide associaation studies
H3ABioNet: H3Africa pan-african bioinformatics network
HPC: High-performance computing
H3Africa: Human health and heredity in africa consortium
NGS: Next generation sequencing
OTU: Operational taxonomic unit
PCA: Principal component analysis
QC: Quality control
SNV: Single nucleotide variants
VCF: Variant call format

## References

[1] Kircher Martin, Kelso Janet (2010). High-throughput DNA sequencing - concepts and limitations. BioEssays.

[2] Sandve Geir Kjetil, Nekrutenko Anton, Taylor James, Hovig Eivind (2013). Ten Simple Rules for Reproducible Computational Research. PLoS Computational Biology.

[3] Schulz WadeL, Durant Thomas, Siddon AlexaJ, Torres Richard (2016). Use of application containers and workflows for genomic data analysis. Journal of Pathology Informatics.

[4] Leipzig J. A review of bioinformatic pipeline frameworks. Brief Bioinform. 2017; 18(3):530–6. 10.1093/bib/bbw020. 10.1093/bib/bbw020 PMC5429012 27013646

[5] Liu Bo, Madduri Ravi K, Sotomayor Borja, Chard Kyle, Lacinski Lukasz, Dave Utpal J, Li Jianqiang, Liu Chunchen, Foster Ian T (2014). Cloud-based bioinformatics workflow platform for large-scale next-generation sequencing analyses. Journal of Biomedical Informatics.

[6] H, 3Africa Consortium. Research capacity. Enabling the genomic revolution in Africa. Science (New York, N.Y.) 2014; 344(6190):1346–8. 10.1126/science.1251546. 10.1126/science.1251546 PMC4138491 24948725

[7] Mulder Nicola J., Adebiyi Ezekiel, Alami Raouf, Benkahla Alia, Brandful James, Doumbia Seydou, Everett Dean, Fadlelmola Faisal M., Gaboun Fatima, Gaseitsiwe Simani, Ghazal Hassan, Hazelhurst Scott, Hide Winston, Ibrahimi Azeddine, Jaufeerally Fakim Yasmina, Jongeneel C. Victor, Joubert Fourie, Kassim Samar, Kayondo Jonathan, Kumuthini Judit, Lyantagaye Sylvester, Makani Julie, Mansour Alzohairy Ahmed, Masiga Daniel, Moussa Ahmed, Nash Oyekanmi, Ouwe Missi Oukem-Boyer Odile, Owusu-Dabo Ellis, Panji Sumir, Patterton Hugh, Radouani Fouzia, Sadki Khalid, Seghrouchni Fouad, Tastan Bishop Özlem, Tiffin Nicki, Ulenga Nzovu (2015). H3ABioNet, a sustainable pan-African bioinformatics network for human heredity and health in Africa. Genome Research.

[8] Amstutz P, Crusoe MR, Tijanić N, Chapman B, Chilton J, Heuer M, Kartashov A, Leehr D, Ménager H, Nedeljkovich M, Scales M, Soiland-Reyes S, Stojanovic L. Common Workflow Language, v1.0. doi.org. 2016. 10.6084/m9.figshare.3115156.v2.

[9] Goecks Jeremy, Nekrutenko Anton, Taylor James, Galaxy Team The (2010). Galaxy: a comprehensive approach for supporting accessible, reproducible, and transparent computational research in the life sciences. Genome Biology.

[10] Kaushik G, Ivkovic S, Simonovic J, Tijanic N, Davis-Dusenbery B, Kural D. Rabix: an Open-Source Workflow Executor Supporting Recomputability and Interoperability of Workflow Descriptions,. Pac Symp Biocomput. 2016; 22:154–65. 10.1101/074708. 10.1142/9789813207813_0016 PMC5166558 27896971

[11] Tang W, Wilkening J, Desai N, Gerlach W, Wilke A, Meyer F. A scalable data analysis platform for metagenomics. In: IEEE international conference on Big Data. IEEE: 2013. p. 21–6. 10.1109/BigData.2013.6691723.

[12] DI Tommaso P, Chatzou M, Floden EW, Barja PP, Palumbo E, Notredame C. Nextflow enables reproducible computational workflows. Nat Biotechnol. 2017; 35:316–9. Nature Publishing Group; Nature Biotechnology, https://www.nature.com/articles/nbt.3820. 10.1038/nbt.3820 28398311

[13] Yang Yaping, Muzny Donna M., Reid Jeffrey G., Bainbridge Matthew N., Willis Alecia, Ward Patricia A., Braxton Alicia, Beuten Joke, Xia Fan, Niu Zhiyv, Hardison Matthew, Person Richard, Bekheirnia Mir Reza, Leduc Magalie S., Kirby Amelia, Pham Peter, Scull Jennifer, Wang Min, Ding Yan, Plon Sharon E., Lupski James R., Beaudet Arthur L., Gibbs Richard A., Eng Christine M. (2013). Clinical Whole-Exome Sequencing for the Diagnosis of Mendelian Disorders. New England Journal of Medicine.

[14] Foo Jia-Nee, Liu Jian-Jun, Tan Eng-King (2012). Whole-genome and whole-exome sequencing in neurological diseases. Nature Reviews Neurology.

[15] Seidelmann SB, Smith E, Subrahmanyan L, Dykas D, Ziki MDA, Azari B, Hannah-Shmouni F, Jiang Y, Akar JG, Marieb M, Jacoby D, Bale AE, Lifton RP, Mani A. Application of Whole Exome Sequencing in the Clinical Diagnosis and Management of Inherited Cardiovascular Diseases in Adults. Circ Cardiovasc Genet. 2017;10(1). 10.1161/CIRCGENETICS.116.001573. 10.1161/CIRCGENETICS.116.001573 PMC5245580 28087566

[16] McKenna A., Hanna M., Banks E., Sivachenko A., Cibulskis K., Kernytsky A., Garimella K., Altshuler D., Gabriel S., Daly M., DePristo M. A. (2010). The Genome Analysis Toolkit: A MapReduce framework for analyzing next-generation DNA sequencing data. Genome Research.

[17] DePristo Mark A, Banks Eric, Poplin Ryan, Garimella Kiran V, Maguire Jared R, Hartl Christopher, Philippakis Anthony A, del Angel Guillermo, Rivas Manuel A, Hanna Matt, McKenna Aaron, Fennell Tim J, Kernytsky Andrew M, Sivachenko Andrey Y, Cibulskis Kristian, Gabriel Stacey B, Altshuler David, Daly Mark J (2011). A framework for variation discovery and genotyping using next-generation DNA sequencing data. Nature Genetics.

[18] Van der Auwera GA, Carneiro MO, Hartl C, Poplin R, del Angel G, Levy-Moonshine A, Jordan T, Shakir K, Roazen D, Thibault J, Banks E, Garimella KV, Altshuler D, Gabriel S, DePristo MA. From fastQ data to high-confidence variant calls: The genome analysis toolkit best practices pipeline. Current Protocols in Bioinformatics. 2013;SUPL.43. 10.1002/0471250953.bi1110s43. NIHMS150003. 10.1002/0471250953.bi1110s43 PMC4243306 25431634

[19] Bolger Anthony M., Lohse Marc, Usadel Bjoern (2014). Trimmomatic: a flexible trimmer for Illumina sequence data. Bioinformatics.

[20] Li H. Aligning sequence reads, clone sequences and assembly contigs with BWAMEM. 2013. arXiv Preprint at https://arxiv.org/abs/1303.3997.

[21] Cingolani Pablo, Platts Adrian, Wang Le Lily, Coon Melissa, Nguyen Tung, Wang Luan, Land Susan J., Lu Xiangyi, Ruden Douglas M. (2012). A program for annotating and predicting the effects of single nucleotide polymorphisms, SnpEff. Fly.

[22] Landrum Melissa J., Lee Jennifer M., Riley George R., Jang Wonhee, Rubinstein Wendy S., Church Deanna M., Maglott Donna R. (2013). ClinVar: public archive of relationships among sequence variation and human phenotype. Nucleic Acids Research.

[23] Nelson Michael C., Morrison Hilary G., Benjamino Jacquelynn, Grim Sharon L., Graf Joerg (2014). Analysis, Optimization and Verification of Illumina-Generated 16S rRNA Gene Amplicon Surveys. PLoS ONE.

[24] McMurdie Paul J., Holmes Susan (2013). phyloseq: An R Package for Reproducible Interactive Analysis and Graphics of Microbiome Census Data. PLoS ONE.

[25] Turner Stephen, Armstrong Loren L., Bradford Yuki, Carlson Christopher S., Crawford Dana C., Crenshaw Andrew T., de Andrade Mariza, Doheny Kimberly F., Haines Jonathan L., Hayes Geoffrey, Jarvik Gail, Jiang Lan, Kullo Iftikhar J., Li Rongling, Ling Hua, Manolio Teri A., Matsumoto Martha, McCarty Catherine A., McDavid Andrew N., Mirel Daniel B., Paschall Justin E., Pugh Elizabeth W., Rasmussen Luke V., Wilke Russell A., Zuvich Rebecca L., Ritchie Marylyn D. (2011). Quality Control Procedures for Genome-Wide Association Studies. Current Protocols in Human Genetics.

[26] Aho AV, Kernighan BW, Weinberger PJ (1987). The AWK Programming Language.

[27] O'Connell Jared, Gurdasani Deepti, Delaneau Olivier, Pirastu Nicola, Ulivi Sheila, Cocca Massimiliano, Traglia Michela, Huang Jie, Huffman Jennifer E., Rudan Igor, McQuillan Ruth, Fraser Ross M., Campbell Harry, Polasek Ozren, Asiki Gershim, Ekoru Kenneth, Hayward Caroline, Wright Alan F., Vitart Veronique, Navarro Pau, Zagury Jean-Francois, Wilson James F., Toniolo Daniela, Gasparini Paolo, Soranzo Nicole, Sandhu Manjinder S., Marchini Jonathan (2014). A General Approach for Haplotype Phasing across the Full Spectrum of Relatedness. PLoS Genetics.

[28] Howie Bryan N., Donnelly Peter, Marchini Jonathan (2009). A Flexible and Accurate Genotype Imputation Method for the Next Generation of Genome-Wide Association Studies. PLoS Genetics.

[29] Ramsay M, Crowther N, Tambo E, Agongo G, Baloyi V, Dikotope S, Gómez-Olivé X, Jaff N, Sorgho H, Wagner R, Khayeka-Wandabwa C, Choudhury A, Hazelhurst S, Kahn K, Lombard Z, Mukomana F, Soo C, Soodyall H, Wade A, Afolabi S, Agorinya I, Amenga-Etego L, Ali SA, Bognini JD, Boua RP, Debpuur C, Diallo S, Fato E, Kazienga A, Konkobo SZ, Kouraogo PM, Mashinya F, Micklesfield L, Nakanabo-Diallo S, Njamwea B, Nonterah E, Ouedraogo S, Pillay V, Somande AM, Tindana P, Twine R, Alberts M, Kyobutungi C, Norris SA, Oduro AR, Tinto H, Tollman S, Sankoh O. H3Africa AWI-Gen Collaborative Centre: a resource to study the interplay between genomic and environmental risk factors for cardiometabolic diseases in four sub-Saharan African countries. Glob Health Epidemiol Genomics. 2016; 1:20. 10.1017/gheg.2016.17. 10.1017/gheg.2016.17 PMC5732578 29276616

[30] Afgan Enis, Baker Dannon, van den Beek Marius, Blankenberg Daniel, Bouvier Dave, Čech Martin, Chilton John, Clements Dave, Coraor Nate, Eberhard Carl, Grüning Björn, Guerler Aysam, Hillman-Jackson Jennifer, Von Kuster Greg, Rasche Eric, Soranzo Nicola, Turaga Nitesh, Taylor James, Nekrutenko Anton, Goecks Jeremy (2016). The Galaxy platform for accessible, reproducible and collaborative biomedical analyses: 2016 update. Nucleic Acids Research.

[31] Wolstencroft Katherine, Haines Robert, Fellows Donal, Williams Alan, Withers David, Owen Stuart, Soiland-Reyes Stian, Dunlop Ian, Nenadic Aleksandra, Fisher Paul, Bhagat Jiten, Belhajjame Khalid, Bacall Finn, Hardisty Alex, Nieva de la Hidalga Abraham, Balcazar Vargas Maria P., Sufi Shoaib, Goble Carole (2013). The Taverna workflow suite: designing and executing workflows of Web Services on the desktop, web or in the cloud. Nucleic Acids Research.

[32] Abouelhoda Mohamed, Issa Shadi, Ghanem Moustafa (2012). Tavaxy: Integrating Taverna and Galaxy workflows with cloud computing support. BMC Bioinformatics.

[33] Ellingson Sally R., Fardo David W. (2016). Automated quality control for genome wide association studies. F1000Research.

[34] Heinzlreiter P, Perkins JR, Torreño O, Karlsson J, Ranea JA, Mitterecker A, Blanca M, Trelles O. A cloud-based GWAS analysis pipeline for clinical researchers. Barcelona: ScitePress; 2014. pp. 387–94. http://www.scitepress.org/PublicationsDetail.aspx?ID=vDrkT5a0WPI=&t=1.

[35] Muniz-Fernandez F., Carreno-Torres A., Morcillo-Suarez C., Navarro A. (2011). Genome-wide association studies pipeline (GWASpi): a desktop application for genome-wide SNP analysis and management. Bioinformatics.

